# Some Uses of Water as a Therapeutic Agent

**Published:** 1893-06

**Authors:** C. C. Stockard

**Affiliations:** Atlanta, Ga.


### SOME USES OF WATER AS A THERAPEUTIC AGENT.*

BY C. C. STOCKARD, M. D.,
ATLANTA, GA.

Water used in various ways has been recognized as of great value in the treatment of disease since the time of Hippocrates, who laid down rules for its use which are followed to this day; but owing largely to the fact that it was taken hold of by quacks, and in the various water-cure and hydropathic establishments advertised to cure every form of disease, it has for a long time been neglected by the profession.

Probably Winternitz, of Vienna, has done more than any other to give modern hydrotherapy the place to which it is entitled in medicine, including as it does the use of every form of water from ice to steam, whether applied to the surface, taken into the stomach by drinking or through a tube; injected into the bowel, vagina or other cavity or under the skin.

Of course the entire subject could not be discussed in a paper such as is expected on an occasion like this, and I shall leave out of consideration the various modes of applying water which cannot be conveniently used except in an establishment especially fitted up for the purpose, and will confine myself to a brief consideration of some of the uses to which it can be readily put in private practice with very great satisfaction to the physician and benefit to his patients.

That property of water which allows it so rapidly to absorb heat from a warmer body and impart it to a colder one, together with

*Read before the Atlanta Society of Medicine, April 18th.

its great cleansing power, make it a therapeutic agent second to none, and I believe there is not one which is so far from being properly appreciated by the profession. It is the so general custom to give medicines in all cases, that our patients are frequently not satisfied with being directed to apply a hot or cold water compress, to wash out the colon with a copious enema, or to wrap the patient in a wet sheet, and it requires some courage to say in a given case that no medicine is needed and advise the simple and harmless measures.

Dr. Hiram Corson, in a letter dated August 16, 1891, to Dr. H. C. Wood, said: "Your paper on the local treatment of dysentery should surely impel others to give it a trial. What you accomplished by the introduction of pieces of ice might, however, be obtained more pleasantly to the patient by the application of ice water cloths over the belly, especially over the course of the lower colon, or with half pint or pint injections of cold water into the rectum. It is amazing to me," continues Dr. Corson, "that so few physicians use cold water as a remedy in inflammatory affections. Of all means of cure in such affections, whenever situated so that the remedy can be applied, there is not one equal to it. In pleuritis, pneumonitis, peritonitis and every other 'itis' it is a most efficient remedy." This, from the pen of a most estimable physician who had used water extensively in a practice of sixty years, is a very strong indorsement.

Dujardin Beaumetz says, "In treating putrid diarrhœa the intestines should be flushed by rectal injections penetrating as far as possible and containing one part in a thousand of naphthol or ten of boric acid", and Blocq, in a recent article on hysteria, says, "The use of cold water once or twice a day by means of the cold affusion, douche or shower, is the most valuable of all external remedies," including electricity, massage, etc. We have probably all of us seen and many felt the delightful effect of an ice water compress to the fevered brow. Many have used with great benefit the same application to the throat in acute inflammation there, and these, with a hot foot bath, warm water emetic or enema and a vaginal douche, make up the sum total of the average doctor's knowledge of hydrotherapeutics.

In the various acute fevers a very ready mode of applying water with great advantage is accomplished thus: Lay on one side of the bed a rubber sheet, a few thicknesses of an ordinary sheet, quilt, blanket or whatever is convenient, to prevent the moisture extending to the bed, and on this lay a sheet wrung out of cold water, so folded that it will reach from the patient's arms to below the knees. Then tuck up the shirt or gown around the neck, and lift or roll the patient on the wet sheet, which is wrapped closely around and covered over with a blanket. At the same time the feet and legs should be wrapped in woolen cloths wrung out of hot mustard water.

While thus wrapped the patient should be well patted or rubbed, exposing a small surface for this purpose at a time, till the entire surface has been gone over. This rubbing and the hot application to the feet keep up a good peripheral circulation or promote rapid reaction, and very much increase the cooling effect upon the entire blood supply, and at the same time lessens the disagreeable sensations of the patient. Where these are dreaded by patients, a little alcoholic stimulant before or during the bath will be serviceable.

These baths may be continued for fifteen or twenty minutes and should be repeated sufficiently often to keep the temperature down, say, whenever it gets over 102°.

In a case of measles, scarlatina, smallpox or typhoid fever, it, together with the free drinking of ice water and an occasional copious cold water enema, will be found sufficient to keep the temperature within bounds, and at the same time proves such a toner for the system generally that the various organs perform their functions well, and we rarely have delirium, deranged stomach, etc., to complicate these cases.

Finding that this paper will be entirely too long drawn out by a consideration of a number of other valuable and easily applied methods of using water, I shall simply say that all will be amply repaid for reading some of the recent works on hydrotherapeutics, of which Dr. Simon Baruch's is an excellent one, and I will devote the remainder of this paper to the injection of water into the rectum or colon, a procedure the real value and the scope of usefulness of which very few physicians have any conception.

About three or four years ago a New York quack advertised extensively a simple and harmless remedy which he had discovered, and which would completely do away with the need for medicines. He sold his little pamphlet divulging the secret for four dollars, and in this short time he has taken in hundreds of thousands of dollars. He advises simply the "flushing of the colon" every second or third day.

We know that in the lower animals the bowels are evacuated many times a day, whereas in man once a day, or even less frequently, is the rule, many going days between evacuations, and I believe that this condition is largely responsible for many chronic diseases.

The absorptive power of the rectum and colon is much greater than is generally thought, and much poisonous matter is reabsorbed which should be promptly expelled on descending to the rectum. It has been stated that iodide of potash introduced into the rectum can be detected in the urine in about twenty seconds and in the saliva in forty.

From this the importance of not allowing fecal matter to collect and remain in the colon can be readily seen, and in chronic diseases especially the washing out of the colon every day or every other day has a better effect than the constant administration of purgatives. The injection of two to four quarts of water will usually be followed by evacuation of any matter lying in the colon, and afterwards by soft actions, showing that peristaltic action has been increased and the contents of the upper bowel is forced down. When cold water has been used there are often several actions within the hour or two following, the latter ones being a good deal like those produced by calomel. Ordinarily it is best to use slightly warmed water.

I think it can be shown to the satisfaction of any who will try, that the kidney's can also be acted on in this way. To accomplish this after the procedure above described, let about one quart of tepid water be injected, and let the patient retire for the night immediately, retaining this quart, and it will be followed by a greatly increased flow of urine, which by washing out the kidneys necessarily removes irritating matters from them, and is of great service in chronic disease of these organs.

In fevers the injection of cold water, if retained for a short while, abstracts very considerable heat, and I have seen a fall of temperature of 1½°, under the tongue, within fifteen minutes, in a case of typhoid fever, follow a large cold water enema.

Besides reducing the fever it has a soothing and beneficial effect upon any inflammatory condition within the abdominal cavity, thus being of genuine service in peritonitis, and when we remember the course of the colon, we see it is like putting an ice bag to the inflamed uterus and appendages, spleen, stomach or liver, and is of great benefit in acute troubles of all these organs.

As for the various disorders of the bowels themselves, the use of enemata is like washing off a wound and ridding it of irritating substances or removing the cinder from the conjunctiva. In dysentery a few injections will usually be found all that is needed if used early, and a thorough washing out, followed by the injection of a solution of zinc sulpho-carbolate, makes an ideal treatment. The same will be found markedly effective in the entero-colitis of children, and I am not sure but that this will be adopted as the most satisfactory treatment of the Asiatic cholera. At least this is the opinion of Dr. Lee, of Chicago, who last summer went to Russia and depending alone upon large injections through a tube introduced well up into the colon, lost only three out of twenty-seven cases treated.

So confident is he of the utility of the method that he has induced Messrs. Tieman & Co. to manufacture the necessary apparatus, and advises the establishment of stations at convenient distances to which patients are to be carried on the first appearance of diarrhœa.

Being unable to more than hint at many matters of importance, without being able to discuss fully any of the methods I have mentioned and leaving entirely out many of great importance, I shall be satisfied if I have aroused sufficient interest in this subject to induce some one to think on and post himself on hydrotherapeutics who has neglected it in the past.

